# Migrant families' access to ECEC and family policies: The Australian and Italian case compared

**DOI:** 10.3389/fsoc.2022.894284

**Published:** 2022-07-22

**Authors:** Manuela Naldini, Elizabeth Adamson, Myra Hamilton

**Affiliations:** ^1^Department of Culture, Politics and Society, University of Turin, Turin, Italy; ^2^Collegio Carlo Alberto, Moncalieri, Italy; ^3^Social Policy Research Centre, Faculty of Arts, Design and Architecture, University of New South Wales, Sydney, NSW, Australia; ^4^Centre of Excellence in Population Ageing Research, University of Sydney, Sydney, NSW, Australia

**Keywords:** migrant families, Italy, Australia, family policies, early childhood education and care

## Abstract

This paper analyses in a comparative perspective the degree of convergence in migrant families' access to early childhood education and care (ECEC) and work/family policies in two different welfare state regimes: Italy and in Australia. Using a framework based on the concept of conditionality—or the notion that access to support is conditional and based on an individual's personal and familial characteristics, circumstances or behaviors—-the analysis examines the extent to which policies designed to support families with young children are accessible to migrant families. It argues that access to ECEC and family policies is restricted in both Italy and Australia according to a series of conditions, but that these conditions apply differently to people of different migrant statuses. In doing so the paper aims to improve our understanding of how welfare states respond to needs associated with migration for children and families and the extent to which they tend to converge.

## Introduction

Families' life trajectories are increasingly characterized by mobility and migration, where life course transitions are often driven by the migration projects of individuals and families. Recently, the social sciences have begun to pay much greater attention to the “importance of the systematic movements of people for work and family life,” known as the “mobility turn” (Sheller and Urry, 2006). However, welfare state studies are yet to adequately engage with the consequences of the mobility turn. In particular, Governments across the developed world are increasingly tightening eligibility requirements and restrictions to access social benefits and entitlements. In addition to workforce activation measures eligibility is increasingly tied to migrant status. Some scholars point out that welfare state pressures have contributed to the rise of sentiment of “welfare chauvinism,” that is, the idea that native citizens are unwilling to grant social rights to foreigners (Freeman, 2009; Mewes and Mau, 2013). Thus, changes to the eligibility requirements to access social benefits are impacting the experiences and opportunities of migrant families. This also has implications for the way that welfare state typologies are constructed and then welfare regimes are compared. Importantly, family benefits and access to ECEC (Early Childhood Education and Care) services offer valuable support for migrant families who often arrive with few resources and limited social networks. Access to family benefits, social rights and ECEC services are critical to support their transition and integration in their new country.

### Restricting migrants' access to social rights

Within the migration literature, opposed to welfare state literature (see Zamora-Kapoor et al., 2017), the scientific debates have long been dominated by questions regarding: the inclusion or exclusion of migrants in receiving countries, implications for citizenship, and access to social benefits and their consequences in term of social inequalities (Sainsbury, 2006; Sainsbury and Morissens, 2012; Boucher, 2014; Anderson et al., 2018). One finding from the migration scholarship is that once immigrants are added into the assessment, the general welfare state typology begins to break down. There has been less attention within the welfare state literature in relation to how non-citizens and migration are integrated into welfare typologies. Sainsbury (2006, p. 23) offers the most developed assessment of how welfare regime types play out in this regard, in so far as she identifies stratification on the basis of immigration visa entry status.

The research that does exist suggests that in many OECD countries, migrant families' access to ECEC and work/care reconciliation policies are constrained by their migrant status (Sainsbury and Morissens, 2012; Shutes, 2016; Westcott and Robertson, 2017; Seeleib-Kaiser, 2018). This is arguably because, through immigration policy and social policies, migrants are restricted from accessing social benefits and entitlements because they do not meet the eligibility requirements. Eligibility is often linked to their visa or residency status, the duration of residency, or other requirements that are more difficult for migrants to meet (i.e., up to date immunisations, secure employment, and so on). This research noted above focuses on immigration policy and entry or residency categories to examine the social rights across regimes.

Thus, literature about families' rights to and utilization of care services in receiving countries remains quite limited Wall and Sao, 2004; Bonizzoni, 2014; Sainsbury, 2019 are exceptions).

### Converging care and migration regimes

Other important contributions have been made by scholars interested in the intersection of care and migration regimes in relation to the social rights, working conditions and inequalities experienced by care and domestic workers (Bettio et al., 2006; Kraler et al., 2011). Diane Sainsbury (2019) points out that this body of literature focuses on the international division of social reproductive labor and immigrant domestic and care work; it is limited in its consideration of migrants' access to and use of care and social benefits. Importantly, recent literature by social policy and care regimes scholars demonstrates that countries with historically different welfare state typologies (UK, Sweden and Spain) are converging with respect to the marketisation of care and the employment of migrant care workers (Williams, 2012). Additionally, a comparative analysis of liberal and Nordic countries' child care regimes reveals that, while there is a convergence in rhetoric, important differences in the detail of the policies remain, which maintain differences in families' access to child care (Mahon et al., 2012). This leads us to ask whether changes in migration and child care policy are leading to convergence or divergence between welfare regimes in terms of migrant families' access to child care and family benefits (Sainsbury, 2006; Williams, 2012).

Sainsbury's cross-national comparison shows the variation in formal rights and substantiate rights, the latter defined as “rights that are operationalized as participation in transfer programs and receipt of benefits” (Sainsbury and Morissens, 2012, p. 1). The analysis reveals the importance of welfare regime type for migrants' substantive rights, and also demonstrates the importance of “entry categories.” Our paper thus aims to make a contribution to this literature through its systematic approach to analyzing the levers, or conditions, that dictate migrants' eligibility to access benefits and services for children and families—-opposed to broader social rights, income maintenance and citizenship. While the experience of marginalization and exclusion varies widely across migrant communities, resulting from intersections between residency status and access to labor markets, access to social rights plays an important role in deepening or ameliorating this marginalization, producing stratifying effects among migrant populations (Kofman, 2002; Morris, 2003; Halevy et al., 2018). Migrant families are therefore likely to benefit considerably from ECEC and work/family reconciliation policies but may be excluded to varying degrees from the welfare state infrastructure through which those policies are made available.

### Immigration policies, welfare state and labor market for migrants in Australia and Italy

Migrants' access to ECEC and work/family policies depends on the migration regimes, welfare state contexts, and labor market contexts in the host country.

This paper focuses on migrant families' access to early childhood education and care (ECEC) and work/family policies in two different welfare state regimes: Italy and in Australia, which have different migration regimes, welfare state types and labor market contexts, as described below.

#### Australia

While Australia has historically favored pathways to permanent residency for migrants, recent changes have shifted the emphasis in Australia's migration regime (Mares, 2016, p. 7). This shift has included a restructuring of visa categories and rules so that there is a much greater emphasis on *skilled* visas (rather than family-based visas) and tighter restrictions on the pathways for visa renewal and transitions to permanent visas status and citizenship (Mares, 2016). Australia has a non-contributory welfare state, which provides means-tested support for eligible parents. Early childhood education and care is subsidized by a combination of federal and state funding: the federal and state governments are committed to providing a preschool program for a minimum of 15 h per week to all children in the year before school, usually at 4 years of age. For younger children, ECEC services are delivered through a market-based system, whereby public funding is attached to the child and flows to a mix of state, community managed and privately managed services (Adamson and Brennan, 2014). Australia has a short period of paid parental leave, 18 weeks paid by the government at the level of the minimum wage, and a set of National Employment Standards which provide employees with statutory paid carers leave (except casual employees) and *unpaid* parental and carers leave, as well as the right to request flexible work arrangements.

Historically, Australia has been described as a 'one and a half earner' model characterized by a high rate of part-time work among women, where ECEC and work/family reconciliation policies are generally organized accordingly. In migrant communities, participation in employment is segregated by gender and residency status: men are more likely to be employed than women, migrants of both genders who are permanent residents/citizens are much more likely than temporary residents to be employed, and are less likely than temporary residents to be in casual or precarious work (ABS, 2017). This is significant because individuals in casual work (i.e., in temporary and irregular jobs) have lower levels of access to leave entitlements. In particular, parents' eligibility to parental leave pay is determined by attachment to the workforce in the year before commencing leave. Participation in casual work also has implications for parents' access to ECEC subsidies, as the number of hours of subsidized ECEC that a family is eligible for is determined by the number of hours worked/fortnight. There is concern by the sector that unpredictable patterns of work are affecting parents' decisions to enroll their children in ECEC because their eligibility for subsidies is unreliable.

#### Italy

In Italy, migrants from European Union (EU) countries enjoy mobility rights and the status of being “EU citizens.” However, even for migrants from within the EU, work-related conditions restrict the right to reside in Italy. To be granted the right to reside in Italy for longer than 3 months (or “legal residency”), EU citizens must be “workers” (or self-employed persons) or have sufficient resources for themselves and their family members not to draw on the social assistance system of the host Members State (see Seeleib-Kaiser, 2018). Only EU citizens who are legal residents (i.e., obtained by being “economically active” or having sufficient resources) can access most non-contributory benefits (Knijn and Naldini, 2018). In contrast, entry by Third Country Nationals (or “TCNs,” citizens from outside of the EU), is bas system of quotas that governs how many TCNs may enter annually, in which industries and from which countries. The quota system has been tightened, and recently frozen, so the pathways for TCNs to migrate to Italy for work are extremely limited. TCNs who reside in Italy uninterrupted for 5 years, have sufficient income and “adequate” accommodation, and have completed a civic integration test, are granted permanent residency, which provides access by TCNs to many non-contributory benefits. Citizenship status is very difficult to obtain even for children of immigrants born in Italy (Caponio et al., 2019).

Italy has a Mediterranean welfare state (Ferrera, 1996) model which combines contributory benefits available to parents with children (i.e., maternity, paternity and parental leaves) with residual social assistance-style means-tested benefits. While ECEC for under-3s is publicly provided, access is very limited and varies hugely geographically. This model of welfare provision is classified as “unsupported familialism” (Saraceno and Keck, 2010), reflective of limited public support for families and high levels of unpaid family care, especially by women. Migrants, both within the EU and TCNs of both genders, are more likely to work in lower-skilled and lower-paid jobs. Migrants often work in ethnicized labor market niches, such as manual work for men (Istat, 2015) and care work for women (Da Roit et al., 2013). There is a higher unemployment rate among migrant women than non-migrant ones, and children born to migrant families are at a higher risk of poverty compared to non-migrants (Saraceno et al., 2013; Santero and Naldini, 2017).

#### Comparing ECEC data and labor markets figures in Australia and Italy

The welfare provisions for families with young children provide important context for understanding the opportunities and constraints for migrant and non-migrant families.

Although public spending for family benefit, as % of GDP, it is higher in Italy than Australia (2.47% and 2.10%, respectively) (OECD, 2019, PF1.1), in 2018 the participation rate in childcare and pre-school services for the very young (0–2 years) was higher in Australia than in Italy (39 and 27.8% respectively). This compares with children aged 3- to 5-years old, where participation is higher in Italy (93.6%) than Australia (84.2%) (OECD, 2019, PF3.2). Maternity and parental leave and provisions that allow parents time to care for children are the second area of family policies crucial for working parents. Comparing Government support for child-related leave, the public expenditure on maternity and parental leave per live birth was 4819.7 USD, in Australia, whereas in Italy the value was almost double at 11060.1 USD (OECD, 2019, PF2.1).

Employment is a core aspect of integration process of migrant people and crucial aspect for having access to welfare state. Comparing labor market outcomes of foreign-born men and women in Italy and Australia we found several differences that reflect diversity among the two labor markets. In Foreign-born women in Australia are much more likely (64%) than foreign-born women in Italy (49.5%) to be employed and, corresponding to this, they are less likely to be unemployed (6.8% compared with 16.2%, respectively). For foreign-born men, the employment rate in the two countries are more similar, at 79.8% in Australia, and 72.4% in Italy (OECD, 2017). However, looking at labor market integration of all foreign-born people compared with native people, we observe a higher employment gap between foreign-born and native population in Australia (-3,4) (which means that foreign-born people are less integrated into the labor market), than in Italy were a positive gap is found (+ 2.3), i.e., foreigner-born people are more likely to be employed than native. This gaps reflect the different distribution of immigrant working population respect to native population in Italy and in Australia. While foreigners in Italy are more like than the native-born population to be active in the labor force (+5.2 points), the foreign-born population in Australia are less likely to be so than the native-born population (-3.3 points). It is also important to point out that in Italy foreign-born people are over-represented among working low-skilled jobs compared with native-born (+22.2 points), while in Australia this indicator is only 1.2 in difference between the two groups (OECD, 2017).

While Australia and Italy show different labor market characteristics, which impact differently on foreign-born men and women employment opportunities, they also have different welfare state types and migration regimes that shape access to ECEC and work/family policies for migrants. Thus, while the two countries share limitations in migrants' integration into the labor market and access to social support to care for children, there are distinct differences in the types and balance of welfare spending and employment across genders and migrant status. In this paper, we ask the question: To what extent can migrant families access ECEC and work/care reconciliation policies in Australia and Italy? In doing so the paper analyses the degree of convergence in migrant families' access, or lack of access, to ECEC and work/family policies in two countries different in terms of welfare state and immigration policy regime.

## Methods and analytical approach

To answer this question, we conducted an audit of eligibility criteria governing access by migrant families to ECEC, family policies and work/family reconciliation policies in Australia and Italy. We organized our analysis into three areas: access to services, access to cash benefits, and access to leave and flexible workplace policies such as parental leave (we label these “services,” “cash,” and “time”). We then conducted an analysis of the policies using a conditionality framework, described below.

The study focuses on families who have migrated on a work or family visa. For the purpose of this paper we have excluded some categories/groups of migrants, namely students (who have usually not formed a family yet), working holiday makers, and families who migrate for humanitarian purposes because the number and proportion of these groups are relatively small in both Italy and Australia and there tend to be eligibility exceptions and targeted supports for migrants entering under the humanitarian stream.

Australia and Italy have been selected following the “most dissimilar country design” (Przeworski and Teune, 1970; Ebbinghaus, 1998), since they have different welfare state types. Following Esping-Andersen (1990) Australia is a liberal welfare state where rights tend to be based on need, and Italy a Mediterranean welfare regime where rights are based on both work and on citizenship. In terms of immigration policy regime (or incorporation regime in Sainsbury's words), Australia is inclusive where rights are based on land of birth, and Italy is restrictive since citizenship status is very difficult to obtain even for children of immigrants born in Italy (Hamilton et al., 2019).

### Analytical approach

In order to disentangle the complex barriers shaping migrants' access to ECEC and work/family reconciliation policies, this paper builds on Clasen and Clegg's (2007) framework of conditionality extended by Shutes (2016) to include migration. The analysis examines the extent to which policies designed to support families with young children are accessible to migrant families. In doing so the paper aims to improve our understanding of how conditions introduced within welfare state policies act to include and exclude migrants from accessing ECEC and work/family policies.

Clasen and Clegg's (2007) developed the framework of conditionality to identify the possible “levels” and “levers” of conditionality that “make access to social benefits [i.e., income support or social security payments] more or less restrictive” in the context of welfare state restructuring (Shutes, 2016, p. 693). Using this framework to identify the way forms of conditionality are introduced in policy across countries and within a country over time, Clasen and Clegg focus on how such forms of conditionality shapes access to social benefits (i.e., income support or social security payments) in the context of welfare state restructuring in the name of activation. Clasen and Clegg distinguish between three “levels” of conditionality, which interact to shape access to social benefits: *conditions of category* (membership of a defined recipient group i.e., people with disability, sole parents); *conditions of circumstance* (eligibility criteria governing access to a benefit i.e., means-test, workforce participation); and *conditions of conduct* (conditions placed on recipients in order to continue receiving the benefit) (Clasen and Clegg's, 2007, p.167). Building on the work of Clasen and Clegg, Shutes (2016) extends the framework of conditionality to include immigration. According to Shutes (2016) the three “levels” of conditionality are interconnected, when considering the ways in which work-related conditionality has intensified in UK policy reform, and they increasingly interact in restricting access to rights of residence/citizenship and to social benefits. Shutes' emphasis in conditions of category is on the role of membership of a defined *migrant* group in shaping access to social benefits.

The frameworks of “work-related conditionality” restricting and governing access to social benefits and access to permanent residence both focus on boosting employment and migrant (perceived) “utility,” especially in economic terms, to the host country (Anderson et al., 2018). The framework of conditionality shaping migration policy reveals a focus on migrants proving that they are employed, employable or economically independent.

In this paper, we build on this framework of conditionality to understand the way in which conditions of category, circumstance and conduct in migration, employment and social policies shape the access of migrant families in Australia and Italy to ECEC and work/care reconciliation policies:

**Conditions of category**: refers to membership of a defined migrant group i.e., temporary residents, permanent residents/citizens, EU citizens, non-EU citizens. Length of residence also creates categories of eligibility for migrant families.

**Conditions of circumstance**: refers to eligibility criteria governing access to ECEC or work/care reconciliation measures such as: individual or household income; workforce participation or employment history; number and/or ages of children; extent of childcare responsibilities (i.e., primary carer); geographical location.

**Conditions of conduct**: refers to conditions placed on recipients in order to continue receiving ECEC or work/care reconciliation measures, such as level of participation in work or training; “good parenting;” and the vaccination of children.

## Results

The audit of ECEC, family policies and work/family reconciliation policies in the two countries are listed in Table 1. The Table presents the ways in which the criteria governing eligibility form conditions of category, circumstance and conduct that, in turn, shape access by migrants to ECEC, family policies and work/family reconciliation policies. The following sections present the analysis for Australia and Italy, followed by a country comparison.

**Table 1:** Comparison of conditions for receipt of ECEC services and benefits for migrant families, Italy and Australia (2020).

		**Italy**	**Australia**
Conditions of category	Services	Public childcare services (0–2 years) Resident requirements Priority to resident parents Non-Eu citizens without long term residence or length of legal residence in the municipality/region may face legal restrictions or practical barriers	Child care subsidy Residency requirements Claimant and their partner must be living in Australia and either: have Australian citizenship; hold a permanent visa; hold a Special Category (humanitarian) visa
	Cash	Child benefit (*Bonus Bebé*) *(under income threshold)* Residency requirements EU citizens with legal residency Non-Eu citizens with a long-term residence permit or refugee status	Parenting payment Residency requirements Claimant and their partner must be living in Australia and either: have Australian citizenship; hold a permanent visa; hold a Special Category (humanitarian) visa Waiting period for new residents Claimants must have been residing in Australia for at least 4 years
	Time	Maternity leave (*contributory based)* Residency requirements None	Unpaid parental leave (non-contributory) Residency requirements None
Conditions of circumstance	Services	Public childcare services (0–2) Work status/history Families where both parents legally employed receive priority access Income circumstances Income test Family circumstances Priority access to single parents, families with disability Subsidy related to household size	Child care subsidy Work status/history Both parents must be working/studying Income circumstances Income test Family circumstances Calculated per child, increases with number of children
	Cash	Child benefit Income circumstances Income tested: household ISEE Claiming provisions Timely application (by 90 days after child's birth)	Parenting payment Work status/history Longer waiting period if you chose to leave your job, were dismissed for misconduct, or moved to an area with lower employment prospects Income circumstances Income and assets tests Family circumstances Need to have a child under 6 (partnered) or 8 years (singles) Increases according to number of children Claimant needs to be the “principal carer”
	Time	Maternity leave: (*contributory based)* Work status/history Employees must have a job at the moment of request. Self-employed mothers required to work during the 12 months before and to have at least 3 months of contributions. Unemployed mothers qualify if the maternity leave starts within 60 days from their last working day	Unpaid parental leave Work status/history Need to have worked with the same employer for at least 12 months; causals need to prove they have worked “regularly and systematically” for their employer for the previous 12 months and are likely to continue working for them into the future Family circumstances Must be the primary carer of the child
Condition of conduct	Services	Public childcare services (0-−2) Parenting practices Immunisations: children must be immunized	Child care subsidy Activity test Must participate in 8–16 h work = 36 h ECEC; 16–48 h = 72 h; 48 + h = 100 h Parenting practices Children need to be fully immunized
	Cash	Child benefit *(Income Tested)* Family circumstances The claimant must not move abroad The claimant must reside with child	Parenting payment Activity test Must participate in job search and job readiness interviews Extensiveness of activity requirements depends on age of child
	Time	Maternity leave: (*contributory based)* Work status/history Must not participate in paid work while on leave	Unpaid parental leave Work status/histroy Must be taken in one continuous period – must remain on leave with exception of 10 “keeping in touch days”

### Australia

In Australia, the following policies were captured in the audit:

- **Services**: Australia provides assistance with the cost of ECEC through a subsidy for families paid directly to the (mostly private) ECEC service (Child Care Subsidy)
- **Cash benefits (“cash”)**: Australia provides tax rebates (Family Tax Benefit (FTB) Part A for low/middle income families, Family Tax Benefit Part B for low/middle income families *with one main earner*) and an income support payment (Parenting Payment) for unemployed parents. It also provides 18 weeks parental leave pay and 2 weeks “partner leave” pay (Parental Leave Pay, Dad and Partner Pay). These sit between cash benefits and leave provisions. While they are payments made during periods of parental leave, they are not formally linked to statutory unpaid leave. Rather, they are administered as if cash benefits through the Department of Human Services (responsible for income support) rather than the Department of Employment (responsible for statutory unpaid parental leave).
- **Leave and flexible workplace policies (time)**: Australia provides unpaid leave following the birth of a child (Unpaid Parental Leave) and paid and unpaid leave when a child is unwell or has an accident (Personal/Carers Leave). It also offers flexible arrangements for parents to balance work and care responsibilities (Right to Request Flexible work)

Access by migrants is shaped by conditions of category, conditions of circumstance, and conditions of conduct.

#### Conditions of category

In Australia, the primary “levers of conditionality” (Clasen and Clegg's, 2007) that govern access by migrants to ECEC and work/family policies are 2-fold: a categorization between temporary residents and permanent residents/citizens (with several notable exceptions); and what are known as the Newly Arrived Resident's Waiting Periods. In most cases, temporary residents as a category are excluded from access to support for services and cash benefits, but not from leave and flexible work provisions, whereas permanent residents/citizens have access to all three sets of provisions. For example, only permanent residents/citizens are eligible for the Child Care Subsidy to support them with the costs of formal childcare, and temporary migrants are *not* eligible (with the exception of some migrant parents on student visas) (Australian Government, 2021a). Eligibility for all other cash payments (FTB A & B, Parenting Payment, Parental Leave Pay, Dad and Partner Pay) require claimants to be permanent residents/citizens. Interestingly, Parental Leave Pay and Dad and Partner Pay are constructed as family assistance payments rather than leave provisions (Baird and Whitehouse, 2012), so are also restricted to permanent residents/citizens.

While permanent residents/citizens are eligible for all of the above services and cash benefits, their access to the payments is also governed by the other condition of category applying specifically to migrants: the Newly Arrived Resident's Waiting Periods. Newly Arrived Resident's Waiting Periods are minimum waiting periods that newly arrived migrants – temporary or permanent – must wait before they become eligible for social benefits (Australian, 2022). Hence, while temporary residents are *excluded* from eligibility for the above provisions and permanent residents/citizens are *included*, permanent residents/citizens must *also* have been residing in Australia for a minimum period to access the provisions. The minimum periods vary for each provision. For example, there is no waiting period for Child Care Subsidy and Family Tax Benefit Part B so permanent residents/citizens are eligible from the time they arrive (provided they meet other conditions of circumstance and conduct). To be eligible for Family Tax Benefit Part A, permanent residents/citizens must wait 1 year, and it is a 2 year waiting period before they become eligible for Parental Leave Pay and Dad and Partner Pay (provided they meet other conditions of circumstances and conduct). The Newly Arrived Resident's Waiting Period for Parenting Payment – the income support payment for unemployed parents – is 4 years.

Access to leave and flexible workplace policies (unpaid parental leave, personal/carers leave, right to request flexible work) are the least conditional provisions for migrant families. There is no Newly Arrived Resident's Waiting Period for these provisions and both temporary and permanent migrants/citizens are able to access these policies, provided they meet certain conditions of circumstance and conduct.

As pathways to permanent residence and citizenship are becoming more difficult to obtain in Australia, and the Newly Arrived Resident's Waiting Periods are gradually being lengthened, the role of conditions of category as “levers” are becoming more restrictive. Combined, these factors are restricting migrants' access to social rights, consistent with Westcott and Robertson (2017) findings.

#### Conditions of circumstance

There are a number of conditions of circumstance governing access to ECEC and work/family policies. Migrants' work status and history is the most important condition of circumstance governing access to services, cash benefits and leave and flexible work provisions. While conditions of category *explicitly* exclude certain groups of migrant families from access, conditions of circumstance associated with work status and history *indirectly* exclude migrants from access due to their differential access to employment opportunities. For example, to be eligible for the Child Care Subsidy, both parents must be participating to some extent in paid work or study (see Conditions of Conduct). In order to be eligible for Parental Leave Pay, the claimant must meet an activity test that requires them to have worked for at least 10 of the 13 months before the birth or adoption of the child (Australian Government, 2021b). This excludes recently arrived migrants, and may also disproportionately affect migrants as they are more likely to have precarious attachment to the labor market. The leave and flexible work provisions – available to both temporary and permanent residents/citizens – are limited in the extent to which they are accessible by some migrants. For example, unpaid parental leave and the right to request flexible work are limited to workers who have been with their employer continuously for 12 months, and casuals are only entitled where they have been working “regularly and systematically” for a period of 12 months and who are likely to continue working for that employer into the future (Fair Work Ombudsman, n.d.). Paid carers leave is not available to casual workers, though unpaid carers leave is available for casual workers. As migrants are more likely to be in casual work (ABS, 2017), and less likely to have been with the same employer for 12 months or more, the limitations on access to leave and flexible workplace provisions are likely to disproportionately exclude migrants.

Other conditions of circumstance include income tests (in some cases designed to “screen in” the needy and in others designed to “screen out” the wealthy (Clasen and Clegg's, 2007), the number and ages of children, and whether the claimant is the primary (or principal carer) of the child. Combinations of these conditions are visible in all of the provisions underpinning services and cash benefits.

#### Conditions of conduct

There has been a general tightening of eligibility requirements for ECEC and family benefits by stipulating the work and care activities that parents must be engaging in to access a payment. This is most evident in the introduction and tightening of activity tests for receiving the Child Care Subsidy, the strict workforce activity/monitoring to receive Parenting Payment, and the increasingly strict ‘parenting' requirements attached to all service support and cash benefits. While these *conditions of conduct* apply to migrants and non-migrants alike, some of the eligibility restrictions create barriers that may be more pronounced for migrants.

For example, the activity test for the Child Care Subsidy that was introduced in July 2018 requires both parents (or a single parent) to be participating in paid work, study or volunteering to receive the Subsidy (Australian Government, 2021b). This provision may disproportionately exclude migrant families from access to the Subsidy, as the proportion of dual earner families is lower in migrant communities. Recently arrived migrant families, in particular, are likely to be excluded from access, as research suggests that secondary visa holders often delay their entry into work and study upon arriving in Australia due to a lack of skill recognition, language, and caring responsibilities (Caponio et al., 2019). At the same time, continuing access to Parenting Payment is subject to increasingly strict job search requirements.

Another condition of conduct for receiving Child Care Subsidy, FTB A & B, and Parenting Payment are immunisations. In order to receive these subsidies and payments, children must be up to date on the National Immunization Program Schedule. It is suspected that recent migrants would be less likely to have an updated record of immunization in Australia, and may therefore be restricted from accessing benefits, even if they have received relevant immunisations in their home countries.

### Italy

In the Italian case the following policies were captured in the audit:

- **Services**: Italy has a 2-fold childcare services system. Childcare services for children under 3 are very limited in coverage and expensive for parents, who must share the cost of the services. Pre-school services for children 3–6 years are considered part of the education system and are therefore fully funded by public administration. Enrolments reach near universality (Alleanza per l'Infanzia, 2020).
- **Cash benefits (cash)**: In Italy, rather than a universal child benefit system, there is a fragmented array of policies to support the cost of children. Three main different types of child benefits can be claimed: a semi-universal cash benefit provided to families below an income threshold (*Bonus bebé*); a means-tested, partially-contributory cash benefit to households (*Assegno al Nucleo Familiare*); and a means-tested transfer targeted to (large) poor families with no waged employment (*Assegno con 3 Figli Minori*). Indirect benefits, i.e., tax allowances are also an increasingly important part of support for the cost of children in Italy (Saraceno, 2017).
- **Leave and flexible work (time)**: Italy, according to Law 53/2000, provides contributory paid maternity leave for 20 weeks at 80 per cent wage replacement, and parental leave for 10 months, of which neither parent can avail for more than six. If the father takes at least 3 months, an extra month is added. A very short paternity leave period has been introduced (Law 92/2012).

#### Conditions of category

In Italy, EU citizens have the right to free movement, whereas entry by migrants from non-EU countries (TCNs) is much more restricted. Once in Italy, access by both EU citizens and TCNs to ECEC, family payments, and work/family benefits depends on their residency status. To be granted the right to reside in Italy for longer than 3 months (or “legal residency”), EU citizens must be employed (or self-employed) and financially self-sufficient. Therefore, for EU citizens the primary distinction is between those who are temporary visitors/residents, and those who are “legal residents.” In contrast, TCNs acquire the right to long term residency if they reside in Italy uninterrupted for 5 years, have sufficient income and “adequate” accommodation, and complete a civic integration test. For TCNs, the primary distinction is therefore between temporary residents and long term residents.

These different residency statuses intersect with the different “welfare logics” (i.e., universal benefits, non-contributory means-tested benefits, contributory benefits) underpinning Italy's ECEC and work/family policies to govern access by migrant families. For example, ECEC services, such as “day-care” or “pre-school services,” are universal, so are accessible to temporary and legal residents (EU citizens), and temporary and long term residents (TCNs). Non-contributory (or partially contributory) means-tested cash and leave benefits are only accessible by legally resident EU citizens and long term resident TCNs. This is consistent with the European Directive 2003/109/EC, which stipulates that long term residents enjoy the same treatment as nationals with regard to social benefits, social assistance and social protection. Contributory leave-based benefits, such as maternity leave, parental leave, and paternity leave, are accessible to temporary and legal residents (EU citizens), and temporary and long term residents (TCNs), provided they have the necessary contributions records (see conditions of circumstance).

#### Conditions of circumstance

Since work-related conditions restrict the access to rights of residence for both for EU citizens and TCNs, and participation in paid work is more important than residency status in shaping access to contributory benefits, conditions of category and conditions of circumstance are closely intertwined.

In Italy, conditions of circumstance – such as participation in formal paid work, adequate income, and for TCNs, adequate accommodation and civic integration – are central to obtaining the residency status (legal residence for EU citizens and long term residence for TCNs) required to access non-contributory (or partially contributory) work/family benefits (funded from general taxation revenue) such as the *Bonus Bebé*, Household Allowance for families with at least three children under 18 years old, or Maternity Allowance (there are two Maternity Allowances: the first (*Assegno di maternità di Stato)* is a partially contributory benefit granted by the State for temporary and ‘precarious' working mothers; the second (*Assegno di maternità municipale*) a means-tested benefit granted by the municipalities for fully unemployed or ‘home-maker' mothers).

Once EU citizens and TCNs obtain the required residency status (legal residence and/ or long-term residence) to be eligible to receive these benefits, they must also meet a series of other conditions of circumstance, such as having an income below a specified level, and meeting requirements about household size and number of children. For instance, the partially contributory Maternity Allowance (*Assegno di maternità di Stato*) also requires that a mother has made 3 months of contributions between 18 and 9 months before the birth.

Participation in paid work is also required in order to build the contributions record needed to access contributory benefits, such as maternity, parental and paternity leave. In the case of these contributory benefits, conditions of circumstance ‘trump' conditions of category, in that there are no distinctions made between EU and TCN citizens when determining the eligibility. Instead, access depends on minimum contribution levels over the 12 months prior to making the claim, which varies depending on the employment status of the parent at the time of claim (i.e., employed, self-employed, unemployed). While both temporary and legal/ long term EU citizens and TCNs are eligible to claim contributory benefits, the minimum requirements regarding contributions records and work histories mean that newly arrived migrants will not have access to these benefits.

Universal benefits such as ECEC services are dependent on income in some circumstances or on family size/characteristics, but not on work status/history.

#### Conditions of conduct

Conditionality of conduct becomes important in governing access to childcare services and pre-school services, where children who are not following the immunization program are excluded from public and private services.

## Discussion

In both Australia and Italy, access by migrants to ECEC and work-family policies is shaped by complex intersections of the conditions of category, circumstance and conduct, especially residency status and/or duration of residence (conditions of category), and labor market status or history (a condition of circumstance and sometimes of conduct). These conditions create varying levels of access by different groups of migrants. The extent to which, and the *way* in which, residency and labor market status/history operate as “levers of conditionality” (Clasen and Clegg's, 2007), and the stratifying effects they have, is linked to the two countries' different migration regimes and welfare regime logics.

In both Italy and Australia, residency status and duration of residence are “levers of conditionality” shaping access by migrants to ECEC and work-family policies. In both countries, eligibility for ECEC and work-family policies differs for two “categories” of migrants: temporary residents and long-term/permanent residents. In Australia, temporary residents are migrants who enter Australia on a temporary visa, and the pathways to permanent residency are varied. It is possible to enter Australia as a permanent resident, usually on a highly-skilled employer-sponsored visa or a family reunion visa. In Italy, temporary residents enter Italy from the EU or non-EU countries for a range of purposes. After 5 years, all temporary residents, provided they have employment and adequate incomes to sustain themselves (and for TCNs, provided they also have adequate accommodation and civic integration) can become “long term residents” or permanent residents. However, after just 3 months, EU citizens who are employed and have adequate income can become “legal residents” whereby they have access to the same social rights as permanent residents/citizens.

In both countries, temporary residents have little or no access to cash benefits (Italy has only one universal cash payment available to both temporary and permanent migrants). In Australia, temporary residents are not eligible for support for ECEC services and in Italy, while access to ECEC services is ostensibly “universal,” permanent residents (and “legal residents”) are accorded priority and temporary residents face some practical barriers to access, such as difficulties registering in their municipality, long waiting lists and access criteria that privilege dual earner families and disadvantage those in irregular work – more likely to be migrants. In contrast, in both countries, permanent residents are eligible for all cash benefits and support for ECEC services (provided they meet other conditions of circumstance and conduct). However, because in Australia it is possible to *enter* in the country as a permanent resident, permanent residents must *also* have been living in Australia for a minimum number of years in order to be eligible for almost all cash benefits. Hence in both Australia and Italy, temporary residents have much more limited access to cash benefits. Temporary residents have no access to ECEC supports in Australia, and in Italy temporary residents are eligible for ECEC services but experience practical barriers. In both countries, permanent residents have access to all ECEC services and cash benefits after a waiting period – in Italy, the waiting period for TCNs is essentially the 5 year period until they are granted permanent residency; in Australia, the waiting period is 1–4 years after arriving in Australia.

In both Australia and Italy, access to leave and flexible workplace policies is much less stratified by residency status than access to ECEC services and cash benefits, and conditions of circumstance become the dominant condition shaping access. In Australia, both temporary and permanent residents are eligible for unpaid parental leave, personal/carer leave, and the right to request flexible work. In Italy, leave provisions are divided into contributory and non-contributory benefits and the contributory benefits are less stratifying according to residency than the non-contributory benefits. In Italy, both temporary and permanent (and legal) residents are eligible for contributory leave provisions (provided they meet other conditions of circumstance and conduct), whereas only permanent (or long term) residents are eligible for non-contributory or partially contributory maternity allowances.

In both countries, residency intersects with employment status and/or history in governing access to ECEC and work/family policies. In Italy, support for ECEC services is not linked to employment status/history. However, employment history intersects with residency status to shape access to leave-based provisions. Contributory leave-based provisions, for which *both* temporary and permanent residents are eligible, require a history of labor market participation and contributions. Non-contributory leave provisions, funded by general revenue, are accessible to permanent residents *only*, and require a shorter contributions history. In Australia, in contrast, support for ECEC services and several cash benefits (all accessible only to permanent residents) *are* linked to participation in employment. This is in part due to Australia's increasing focus on the “activation” of cash benefit recipients. Leave-based provisions, accessible to *both* temporary and permanent migrants and funded by employers, require at least 12 months working with the same employer and are more difficult to access for casual employees. Parental leave pay (which sits between a cash benefit and a leave policy in Australia) is funded by general revenue, is accessible to permanent residents *only*, and requires ~12 months labor market participation. In sum, in all cases, where provisions are contributory or not funded by general revenue, residency status or length of residency are less important in governing access to provisions. However, access to these provisions *is* governed by employment history and/or contributions record (something that newly arrived residents are less likely to have achieved). In Australia, unlike Italy, some general-revenue-funded provisions (available only to permanent residents) are also governed by employment status or history. In most cases, the levels of conditionality (especially residency and employment history) require that the migrant family has lived and/or worked in the new host country for a minimum period of time in order to be eligible for work/family policies. Access to ECEC and work/family policies immediately after migration would have the potential to act as a “buffer” for migrant families as they navigate some of the risks that are either specific to migration and mobility or heightened by migration and mobility, such as employment transitions, precarious work, lack of recognition of skills (or human capital depletion), social deprivation and exclusion, and difficulty balancing paid work and unpaid care.

Instead, these levels of conditionality intersect with poor labor market opportunities for migrants, and gender and cultural norms in migrant families, to deepen legal and institutional barriers to migrant parents' access to measures designed to support families with children and to help parents reconcile work and care responsibilities. Migrants who are more marginalized from labor markets are in the greatest need of support, yet often, it is strong attachment to employment that creates pathways to permanent residency and all of the access to ECEC and work/family policies that come with that.

## Conclusion

The findings and discussion above demonstrate key differences in Australia and Italy's welfare and immigration regimes, particularly in the logic of the restrictions to access ECEC services, family cash benefits and leave entitlements. One of the most striking difference is linked to Italy's distinction between EU and non-EU migrants and its impact on the application of temporary vs. permanent/legal immigrants. That is, EU immigrants need only be living in Italy for 3 months to gain legal status, compared with TCNs who must have lived in Italy for 5 years to gain residency status. Australia distinguishes primarily as temporary and permanent residency, however even permanent residents must meet minimum waiting periods. Despite categorical differences in entry requirements, definitions and waiting periods for temporary and permanent migrants, there are convergences in the way they are increasingly contingent on labor force attachment.

The findings also demonstrate a level of convergence in regard to conditions of circumstance. This is most apparent with respect to access to cash benefits. Despite different welfare and immigration logics, in both countries access to cash benefits is restricted by conditions of circumstance, predominantly parents' attachment and contributions to the labor market. While access to ECEC services is a universal provision in Italy, and therefore conditions of circumstance and conduct do not in principle impact eligibility, practical barriers and municipal resources means this is not a reality for many immigrant families. This compares with Australia where recent changes have imposed strict conditions of circumstance and conduct to access subsidies for ECEC. Conditions of conduct are much less salient for Italy, where it is only relevant for children's immunization and access to preschool services. This compares with Australia where conditions of conduct are becoming more important for parents, particularly single mothers, as a condition for accessing benefits is staying meeting strict activity tests and having up to date immunization records. In addition to these concrete conditions and restrictions placed on immigrant families, we must also remember that migration and mobility increase the vulnerability for families in other ways, such as precarious work, family breakdown and capacity to balance work and care, and creates other risks, such as human capital depletion.

The analysis in this paper suggests that migration may entail exposure to another, as yet overlooked risk associated with migration: a temporary deficit of social rights. Amplifying this risk is the policy tendency in both Australia and Italy to increase conditionality in access to permanent residency and to social rights, extending the duration and nature of this deficit.

The findings demonstrate that migrant families' social rights are affected by welfare regime type especially in terms of access to ECEC services. However, the analysis shows also that a convergence between the two countries is emerging, particularly the importance of immigration regimes imposing conditions for accessing to family cash benefits for migrants who – by definition or duration – are deemed temporary migrants. Overall, the findings illustrate the way that migration policies – and related conditions on access to benefits and services – disrupt traditional assumptions about welfare regimes and access to social rights. Regardless of regime type, the findings show that countries with different immigration regimes and logics of residency and employment are increasingly implementing policies that impose conditions for migrant families to access cash benefits, ECEC services and leave entitlements.

## Data availability statement

The raw data supporting the conclusions of this article will be made available by the authors, without undue reservation.

## Author contributions

MN, EA, and MH contributed to conception, design of the study, and wrote paragraphs 4 and 5. MN and MH wrote the first draft of the manuscript of paragraph 1, 2. EA wrote paragraph 3. All authors contributed to manuscript revision, read, and approved the submitted version.

## Funding

This work was supported by the Australian Research Council, through ‘Markets, Migration and the Work of Care’ [DP160100175].

## Conflict of interest

The authors declare that the research was conducted in the absence of any commercial or financial relationships that could be construed as a potential conflict of interest.

## Publisher's note

All claims expressed in this article are solely those of the authors and do not necessarily represent those of their affiliated organizations, or those of the publisher, the editors and the reviewers. Any product that may be evaluated in this article, or claim that may be made by its manufacturer, is not guaranteed or endorsed by the publisher.
